# Draft genomes of multidrug-resistant *Providencia manganoxydans* strains isolated from shrimp (*Penaeus monodon*) in Bangladesh

**DOI:** 10.1128/mra.00391-25

**Published:** 2025-08-11

**Authors:** Md. Iftehimul, Muhammad Tofazzal Hossain, Mohammad Mahfujul Haque, Neaz A. Hasan

**Affiliations:** 1 Department of Biotechnology, Bangladesh Agricultural University 54492, Mymensingh, Bangladesh; 2 Department of Microbiology and Hygiene, Bangladesh Agricultural University 54492, Mymensingh, Bangladesh; 3 Department of Aquaculture, Bangladesh Agricultural University 54492, Mymensingh, Bangladesh; 4 Department of Fisheries and Marine Bioscience, Gopalganj Science and Technology University 823103, Gopalganj, Bangladesh; California State University San Marcos, San Marcos, California, USA

**Keywords:** draft genome sequence, *Providencia manganoxydans*, *Penaeus monodon*

## Abstract

*Providencia manganoxydans is* gaining interest in aquaculture for its manganese-oxidizing function, with possible roles in detoxifying water and enhancing shrimp health. We initially report the genome sequencing of *P. manganoxydans* strains BD001 and BD002 (4.7 Mbp each) isolated from shrimp, with 40.7% and 40.0% GC contents, and 80× and 130× sequencing depths, respectively.

## ANNOUNCEMENT

Shrimp aquaculture is a developing global industry with a multiform set of farming systems (1–6) that are all impacted by various microbial communities (7–10). Both Gram-negative and Gram-positive bacteria frequently afflict such systems and either positively or negatively influence shrimp health and production (11).* Providencia manganoxydans* is a Gram-negative, manganese-oxidizing bacterium that has gained interest in shrimp aquaculture and has potential role in improving water quality and promoting shrimp health (12, 13). It converts soluble Mn(II) into insoluble Mn(IV), helping detoxify manganese-rich environments common in intensive aquaculture systems (14), reduces mineral stress, and limits harmful heavy metals bioavailability, which negatively impact shrimp (12). Although it has been less studied than traditional probiotics, unique manganese-oxidizing function highlights its potential as microbial tool for environmentally friendly shrimp farming practices.

*P. manganoxydans* was isolated from *Penaeus monodon* intestine, collected from a shrimp farm in Khulna, Bangladesh. Shrimp were dissected under sterile conditions, and intestinal tracts were aseptically removed using sterile forceps. The pooled intestinal samples were homogenized and enriched in Brain Heart Infusion (BHI, Oxoid, UK) broth supplemented with 2% NaCl. After overnight incubation at 37°C, enriched cultures were plated on Thiosulfate-Citrate-Bile Salts-Sucrose agar (TCBS, Oxoid, UK), yielding pure colonies (15). Identification of *P. manganoxydans* was done based on its characteristic colony morphology (yellowish, round shape) and Gram-negative staining (16). Species identification was further confirmed using the VITEK-2 System (v9.01) (17). Antibiotic susceptibility testing (AST) was performed using the disc diffusion method, following the CLSI and EUCAST 2024 guidelines (18). Genomic DNA was extracted using the GeneJET Genomic DNA Purification Kit (Thermo Fisher Scientific, USA) from the cultured broth, and quantity and purity were measured with Nanodrop (Thermo Fisher Scientific, USA). A DNA library was prepared using Nextera XT Library Prep Kit (Illumina, San Diego, CA, USA), and sequencing was performed on Illumina MiSeq platform with UDPO101 adapters, where strain BD001 produced 1.3 million paired-end reads (376.3 million bases), and strain BD002 generated 2.1 million paired-end reads (613.4 million bases). Quality control of the raw reads was conducted using FastQC v0.12.1 (19), and low-quality sequences were trimmed using Fastp v0.24.0 (20). The high-quality reads were assembled into *de novo* genomes using SPAdes v4.0.0 (21), and the assembly was evaluated with QUAST v5.3.0 (22). Genome annotation was performed with NCBI Prokaryotic Genome Annotation Pipeline (PGAP) v6.9 (23), and genome completeness and contamination were assessed using CheckM v1.2.3 (24), and ANI was calculated using the OrthoANI algorithm via the EZBioCloud (25). Functional annotation and antibiotic resistance genes (ARGs) were identified using ResFinder (26), plasmids with PlasmidFinder (27), and virulence factors (VFGs) through VFDB (28), bundled in ABRicate v0.8.13. Subsystem analysis was carried out with the RAST v2.0 server (29), and pathogenicity was evaluated using PathogenFinder v1.1 (30). Additionally, bacteriocin gene clusters, prophages, and MGEs were identified with BAGEL4, PHASTEST v1.0.1, and ICEFinder v2.0, respectively (31–33). All genomic analyses were performed using default parameters.

Genomic characteristics of *P. manganoxydans* strains (BD001 and BD002) have been summarized in Table 1. Both genomes are approximately 4.7 Mbp in size, with BD001 showing a guanine–cytosine content of 40.7% and BD002 slightly lower at 40.0%. The sequencing coverage was 80× for BD001 and 130× for BD002. Both strains showed 98.74% genome completeness with 3.51% contamination, each carrying six ARGs, one plasmid, and MGEs (four in BD001, three in BD002), but no detectable VFGs (34). The number of subsystems associated with various metabolic functions was consistent at 25 in both strains (34). Interestingly, both strains had a moderate pathogenicity score of 0.518 and matched with 10 known pathogenic families, suggesting a potential but limited pathogenic capacity. However, AST revealed that BD001 was resistant to 14 antibiotics (4 intermediate), and BD002 to 13 antibiotics (5 intermediate), spanning 10 drug classes. Both strains demonstrated complete (100%) sensitivity to oxytetracycline, doxycycline, tetracycline, levofloxacin, and chloramphenicol, underscoring the multidrug-resistant (MDR) nature of both strains (35). These findings highlight the MDR potential of *P. manganoxydans* strains, offering important insights into their genomic architecture and raising considerations for their environmental and clinical relevance.

**TABLE 1:** Genomic features of the *P. manganoxydans* strains isolated from *P. monodon*

Strain	*P. manganoxydans *strain BD001	*P. manganoxydans s*train BD002
GenBank accession	JBMKKI000000000	JBMKKH010000000
BioSample accession	SAMN47584801	SAMN47584802
SRA accession	SRX28154487	SRX28154488
N50 value (kb)	148.8	138
Number of contigs	133	135
Largest contig (bp)	339,369	339,369
Average Nucleotide Identity (ANI) (%) compare to RefSeq: GCF_016618195.1	98.73	98.75
Genes (total)	4,419	4,411
CDSs (total)	4,341	4,336
Genes (coding)	4,275	4,270
CDSs (with protein)	4,275	4,270
Genes (RNA)	78	75
rRNAs	1, 6, 9 (5S, 16S, 23S)	3, 4, 6 (5S, 16S, 23S)
Complete rRNAs	1 (5S)	3 (5S)
Partial rRNAs	6, 9 (16S, 23S)	4, 6 (16S, 23S)
tRNAs	55	53
ncRNAs	7	9
Pseudo genes (total)	66	66
CDSs (without protein)	66	66
Number of intact prophages	3	3
Number of bacteriocins	2	2

## Data Availability

The raw genome sequence data of *P. manganoxydans* were submitted to NCBI, GenBank, and SRA under BioProject number PRJNA1242270.
